# Effects of 12 weeks of complex training on lower limbs strength and power in collegiate dancers

**DOI:** 10.7717/peerj.20486

**Published:** 2026-05-21

**Authors:** Yang Lei, An HyoSun, Kaimei Xu, Junsheng Cao

**Affiliations:** 1 ChangZhou Vocational Institute of Industry Technology, Changzhou, China; 2 Hanyang University, Seoul, South Korea; 3 Wenshan University, Wenshan, China; 4 ShanDong Normal University, Jinan, China

**Keywords:** Complex training, Collegiate dancers, Lower limb strength, Resistance training, Plyometric exercises

## Abstract

**Background:**

Complex training (CT), which combines resistance and plyometric exercises within a single session, is widely used to improve strength and explosive performance. However, no study has examined its effectiveness among collegiate dancers, who require both strength and dynamic control for performance. This study aimed to compare the effects of 12 weeks of CT versus traditional resistance training (RT) on lower-limb strength and power in collegiate dancers.

**Methods:**

Thirty-six collegiate dancers were randomly assigned to either a CT group (*n* = 18) or an RT group (*n* = 18). Both groups trained twice weekly for 12 weeks under supervision. Performance outcomes included squat jump (SJ), countermovement jump (CMJ), isometric mid-thigh pull (IMTP), specific anaerobic performance (PSAP), reactive strength index (RSI), and one-repetition maximum (1RM) squat. Pre- and post-training tests were analyzed using two-way repeated-measures ANOVA.

**Results:**

After the intervention, the CT group showed significantly greater improvements than the RT group in CMJ (Δ + 19.4%, *d* = 0.86), PSAP (Δ + 31.6%, *d* = 0.81), and RSI (Δ + 7.2%, *d* = 0.58) (all *p* < 0.01). Both groups demonstrated significant increases in SJ, IMTP, and 1RM squat performance (all *p* < 0.001), with the CT group exhibiting slightly larger effect sizes (SJ: *d* = 0.84; IMTP: *d* = 0.72; 1RM: *d* = 0.65).

**Conclusions:**

These findings suggest that CT could be an effective training method for enhancing the physical performance of collegiate dancers, potentially benefiting their technical and aesthetic dance skills. Further research should explore the applicability of these findings to male dancers and other dance styles.

## Introduction

Dance is a physically demanding art form that integrates artistic expression and athletic prowess. To execute complex movements with precision and grace, dancers must possess exceptional strength, power, flexibility, and endurance (Brown et al., 2007). Among these attributes, lower limb strength and power are particularly crucial because they underpin key techniques such as jumps, leaps, and turns (Ambegaonkar et al., 2018). Collegiate dancers face additional challenges due to their intensive training and performance schedules. They must balance their academic commitments with rigorous dance training, frequently attending multiple classes and rehearsals each day (Sanders et al., 2021). Such a demanding schedule can result in overuse injuries and fatigue, particularly in the lower extremities (Huang et al., 2022). Consequently, the development of effective training strategies to enhance dancers’ physical capabilities is essential.

One modality that has attracted considerable attention in sports and dance performance is complex training. Complex training entails alternating heavy resistance exercises with plyometric drills within a single session (May, Cipriani & Lorenz, 2010; Liu et al., 2022). This approach exploits post-activation potentiation (PAP), whereby a preceding high-intensity exercise transiently enhances subsequent explosive movements (Drouzas et al., 2020). Plyometric training, a core component of complex training, has been shown to improve jumping ability and aesthetic performance in female collegiate dancers (Brown et al., 2007). Although complex training is widely studied in athletes, no research has explored its effects in collegiate dancers.

The distinctive demands of dance necessitate a balance between strength and power development and the preservation of movement aesthetics. Complex training may meet this challenge by coupling heavy-resistance exercises that build strength with plyometric exercises that enhance power and explosiveness of the lower limbs. Such a combination could improve dance-specific skills, such as jumps and leaps, without compromising the artistic elements. Moreover, the time-efficient nature of complex training is particularly advantageous for collegiate dancers who must judiciously allocate limited training time. Integrating strength and power exercises within a single session may yield substantial physical gains without adding excessive volume to demanding schedules (Sanders et al., 2021).

However, implementing complex training in dance contexts requires careful consideration. Dancers frequently express concerns about the potential adverse effects of strength training on their physique and flexibility (Rosenthal et al., 2021). Therefore, strength and power programs must complement dancers’ existing regimens and aesthetic requirements. Additionally, the physiological adaptations elicited by complex training in dancers may differ from those in other athletic cohorts. Dancers’ distinctive movement patterns, coupled with the importance of flexibility, may modulate training outcomes.

In light of these considerations, the present study investigated the effects of a 12-week complex training program on lower-limb strength and power in collegiate dancers. We hypothesized that CT would produce greater improvements in explosive power (countermovement jump (CMJ), reactive strength index (RSI), specific anaerobic performance (PSAP)), than RT, while both would improve maximal strength equally.

## Materials and Methods

### Participants

**Figure 1:**
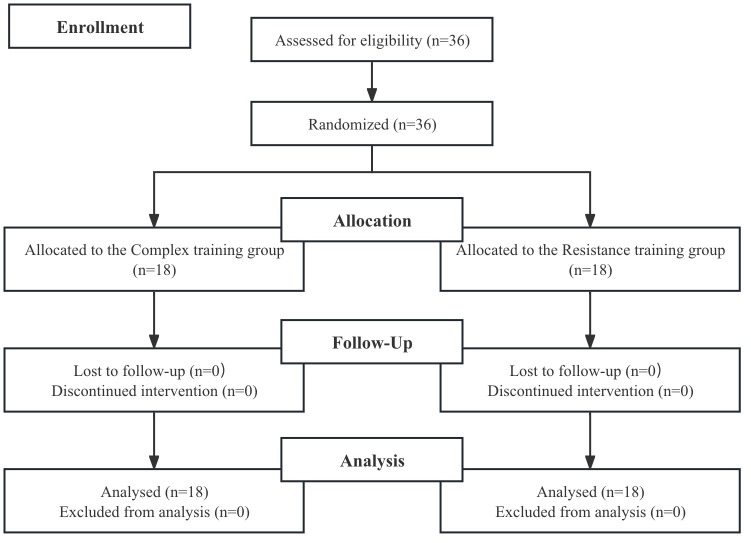
Flow chart of the progress through the phases of the study according to the CONSORT statements.

This study involved 36 male collegiate dancers, recruited between March 9, 2024, and April 9, 2024, from a local university (Fig. 1). All participants were enrolled in a modern dance program but had prior experience in other dance styles, including jazz and hip-hop. None had formal resistance training experience before the study. The study employed an unblinded pre-post interventional design, with assessments focusing on anthropometric measures (height and body mass), maximal lower-limb strength and power, and performance indicators, including countermovement jump (CMJ), squat jump (SJ), pre-stretch augmentation percentage (PSAP), reactive strength index (RSI), 1RM squat, and isometric midthigh pull (IMTP).

 Participants, aged 18–23 years, were eligible if they could commit to all testing and training sessions and had no history of severe lower-body injuries (including anterior cruciate ligament, hamstring, meniscus, or ankle injuries) in the past three years. Additionally, participants could not have any medical or orthopedic conditions that might interfere with their involvement in the study. Prior to participation, all dancers were fully informed about the study procedures, potential risks, and benefits, and written informed consent was obtained. The study was approved by the Shandong Normal University Ethics Committee (Approval number: 2024136) and adhered to the latest revision of the Declaration of Helsinki.

### Procedures

All participants underwent a 2-week adaptation strength training program, consisting of 3 sessions per week. The purpose of this program was to familiarize participants with strength training and the exercises incorporated in the subsequent program. Each participant used a 20 kg empty barbell during the training. The aim was to optimize exercise technique, minimize the risk of injury, and prepare the participants for the upcoming training regimen. The exercises were selected based on the muscle groups engaged during dance, and strength exercises were chosen to complement the subsequent training program (Koutedakis & Jamurtas, 2004).

**Figure 2:**
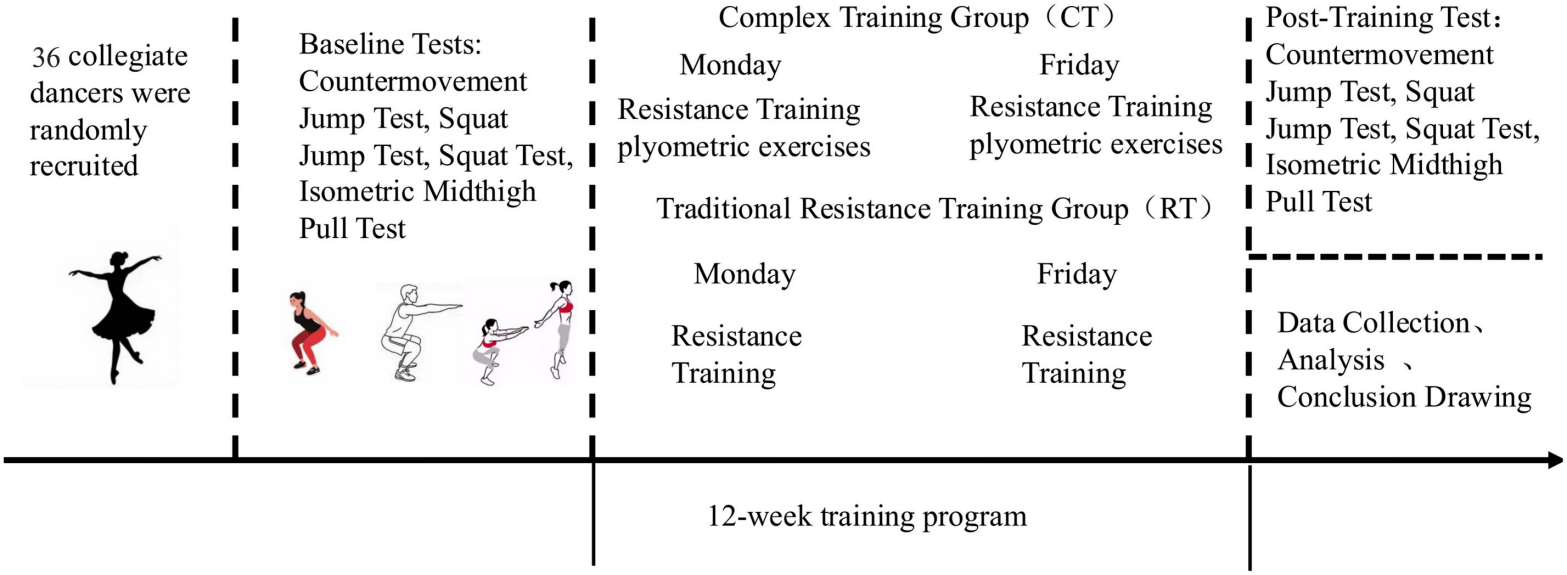
The timeline and session structure.

After completing the adaptation period, all participants underwent baseline evaluations, including the SJ, CMJ, RSI, PSAP, IMTP, and 1RM squat tests (Fig. 2). Participants were then randomly assigned to either the complex training (CT, *n* = 18) or resistance training (RT, *n* = 18) groups using a computer-generated randomization list (SPSS, IBM Corp., Armonk, NY, USA; Table 1).

**Table 1:** Physical characteristics of participants in the CT and RT groups.

	Age (y)	Height (cm)	Mass (kg)	BMI (kg m^2^)
CT (*n* = 18)	21.00 ± 1.58	177.53 ± 2.95	69.04 ± 2.77	21.90 ± 0.47
RT (*n* = 18)	20.00 ± 1.72	174.91 ± 5.05	67.72 ± 3.98	22.07 ± 0.94

Testing was conducted approximately one week before and after the 12-week training program, at consistent times of day, with all tests completed within a single session (∼60–75 min). The order of tests was standardized as follows to minimize fatigue effects: SJ → CMJ → Drop Jump/RSI → IMTP → 1RM back squat. Two minutes of passive recovery were allowed between trials of the same test and 3–5 min between tests. Before each jump test, participants completed two submaximal familiarization trials at 50–70% perceived effort.

All training and testing sessions were supervised by experienced strength and conditioning staff. Each session included standardized warm-up and cool-down procedures, which are described in Supplemental Information 1.

### Measurements

Participants were instructed to avoid any intensive physical activity for at least 48 h prior to testing. They were also asked to consume food within 4 h before the assessments. A 5-minute warm-up was conducted prior to the tests, consisting of low-intensity running, high skipping, leg flexions, lateral running, arm rotations (front and behind), and sprints. Following the warm-up, participants performed 5 min of stretching exercises. To ensure familiarity with the assessments, participants completed 1–3 practice trials of each test.

#### 1RM strength test

The 1RM back squat test was conducted in a power rack (Hammer Strength, Rosemont, IL, USA) using a previously described protocol (McBride et al., 1999). After the general warm-up, participants completed four preparatory sets: 10 repetitions at 50%, five at 70%, three at 80%, and one at 90% of their estimated 1RM. The estimated 1RM, calculated from a submaximal set (≤10 repetitions at a self-selected load) using the Epley equation (1RM = load × [1 + reps/30]), was used solely to prescribe these warm-up loads. Subsequent to the warm-up, participants attempted to establish their actual 1RM within three to four trials, with 3–5 min of rest between efforts. For each load, up to two attempts were permitted. Squat depth was standardized to a parallel squat (≈90° knee flexion, thighs parallel to the ground), verified by a lateral rater. Two spotters supervised all maximal attempts to ensure safety.

#### IMTP test

The isometric midthigh pull (IMTP) is a reliable and efficient method for assessing lower limb strength in adolescents (Till et al., 2018). Prior to testing, the mid-thigh position was marked for each participant by identifying the midpoint between the knee and hip joints. Participants were instructed to assume their preferred deadlift position, selecting their hip and knee angles. The height of the barbell was adjusted to ensure it was in contact with the mid-thigh. Participants were allowed to use an overhand, mixed, or hook grip. Upon receiving the command “GO!”, participants were instructed to pull upward on the barbell as hard and as fast as possible, maintaining maximal effort for 6 s. To prevent precontraction, participants were asked to relax before the “GO!” command. The force-time curve for each trial was recorded using a force plate (Kistler 9281CA, KISTLER, Winterthur, Switzerland) at a sampling rate of 1,000 Hz (Comfort et al., 2015). Peak force was defined as the highest force achieved during the 6-second isometric effort, minus the participant’s body weight in Newtons.

#### CMJ and SJ test

The vertical jump tests included the CMJ and SJ, following established protocols (Markovic et al., 2004). For each jump test, participants completed three trials with a 2-minute recovery between them, with the highest performance used for further analysis. Prior to the tests, two submaximal practice trials were performed, with 1-minute recovery. The specified 2-minute recovery refers to rest between trials of the same test; a 3–5 min passive recovery was provided between different tests within the session.

For CMJ, participants used a self-selected countermovement depth with hands on hips to preserve ecological validity; technique was reinforced during familiarization and practice trials to promote within-subject consistency and avoid knee flexion exceeding ∼90°. For SJ, depth was standardized at 90° knee flexion held for 3 s prior to take-off (Smart Jump; Fusion Sport, Brisbane, Australia).

#### PSAP test

Prior to the Drop Jump trials, participants performed two submaximal practice drop jumps (50–70% effort) after the general warm-up to ensure technique familiarity. A 30 cm box height was selected as a commonly adopted, standardized stimulus for assessing reactive strength while balancing SSC intensity and safety in novice-to-intermediate cohorts; this height is widely used in RSI protocols and reduces between-participant variability in ground contact times and countermovement strategies. The PSAP and RSI were used to indirectly assess an athlete’s ability to utilize the stretch-shortening cycle (SSC) to enhance jump height and peak power during a vertical jump, which is commonly used as an indicator of lower-limb power performance (Suchomel, Sole & Stone, 2016). The PSAP index was calculated from the jump data as follows:

\begin{eqnarray*}\mathbi{PSAP}= \frac{\mathbi{CMJ~ height}-\mathbi{SJ~ height}}{\mathbi{SJ~ height}} \times 100\% \end{eqnarray*}

Reactive strength was measured using drop jump (DJ) height and the RSI (McBride et al., 1999). For the DJ test, participants stood on a 30 cm-high box with their hands on their hips (Pedley et al., 2022). They then stepped off the box, landing on the force plate, and performed a vertical jump for maximum height with minimal ground contact time. A trial was considered successful if participants did not bend their hips or knees during the jump and kept their hands on their hips. Three trials were conducted, separated by 1 min of passive recovery, and the best jump height was used for data analysis. RSI was calculated by dividing jump height (mm) by contact time (ms) (Barker, Harry & Mercer, 2018).

\begin{eqnarray*}\mathbi{RSI}= \frac{\mathbi{jump~ height}~(\mathbi{mm})}{\mathbi{ground~ contacttime}~(\mathbi{ms})} . \end{eqnarray*}

### Training program

Participants in the CT group trained twice weekly (Mondays and Fridays) for 12 weeks, with each 60-min session structured into three progressive 4-week stages. Following established protocols for novices (Kraemer et al., 2002), the training program comprised five complex pairs combining resistance and plyometric exercises relevant to dance movement patterns. Each pair was performed for three sets with 3-min intra-pair and 4-min inter-pair rest intervals, consistent with recommendations for optimizing PAPE and strength gains (Mina et al., 2019; Suchomel et al., 2018).

Participants in the RT group completed the same 12-week RT program, performed on the same days as CT. On each training day, participants completed the resistance training movements outlined in Tables 2 and 3. Training intensity began at approximately 60% of each participant’s 1RM during weeks 1–4 and increased to 65–70% 1RM during weeks 5–8, and 80% 1RM during weeks 9–12.

**Table 2:** Complex training program.

Exercises	Stage 1 (Week 1–4)	Stage 2 (Week 5–8)	Stage 3 (Week 9–12)
Complex pair 1	Back Squat (60%1RM × 8–12RM × 2sets) Vertical Jump (8–12RM × 2sets)	Back Squat (65–70%1RM × 8–12RM × 3sets) Box Jump (8–12RM × 3sets)	Back Squat (80%1RM × 6–12RM × 2–4sets) Drop Jump (6–10RM × 2–4sets)
Complex pair 2	Hexagonal Barbell Pull-up (60%1RM × 8–12RM × 2sets) Broad Jump (8–12RM × 2sets)	Hexagonal Barbell Pull-up (65–70%1RM × 8–12RM × 3sets) Squat Jump (8–12RM × 3sets)	Hexagonal Barbell Pull-up (80%1RM × 6–12RM × 2–4sets) Drop Jump (6–10RM × 2–4sets)
Complex pair 3	Weight-bearing Lunge (60%1RM × 8–12RM × 2sets) Split-leg Squat Jump (8–12RM × 2sets)	Weight-bearing Lunge (65–70%1RM × 8–12RM × 3sets) Single-legged Side Box Jumps (8–12RM × 3sets)	Weight-bearing Lunge (80%1RM × 6–12RM/leg × 4–8sets) Single-legged Drop Jump (6–10RM/leg × 2–4sets)
Complex pair 4	Weight-bearing Heel-lifting (60%1RM × 8–12RM × 2sets) Jump on Tiptoe (8–12RM × 2sets)	Weight-bearing Heel-lifting (65–70%1RM × 8–12RM × 3sets) Jump on Tiptoe (8–12RM × 3sets)	Weight-bearing Heel-lifting (80%1RM × 6–12RM × 2–4sets) Jump on Tiptoe (6–10RM × 2-4sets)
Complex pair 5	Leg Press (60%1RM × 8–12RM × 2sets) half-squat jump (8–12RM × 2sets)	Leg Press (65–70%1RM × 8–12RM × 3sets) Squat jump (8–12RM × 3sets)	Leg Press (80%1RM × 6–12RM × 2-4sets) Box Jump (6-10RM × 2-4sets)
Rest	Pairs:3 min rest interval Between groups:3-4 min rest	Pairs:3 min rest interval Between groups:3-4 min rest	Pairs:3 min rest interval Between groups:3-4 min rest

**Notes.**

RM: maximum repetitions; % 1RM, percentage of 1RM maximum load intensity.

**Table 3:** Resistance training program.

Exercises	Stage 1 (Week 1–4)	Stage 2 (Week 5–8)	Stage 3 (Week 9–12)
Complex pair 1	Back Squat (60%1RM × 8–12RM × 2sets)	Back Squat (65–70%1RM × 8–12RM × 3sets)	Back Squat (80%1RM × 6–12RM × 2-4sets)
Complex pair 2	Hexagonal Barbell Pull-up (60%1RM × 8–12RM × 2sets)	Hexagonal Barbell Pull-up (65–70%1RM × 8–12RM × 3sets)	Hexagonal Barbell Pull-up (80%1RM × 6–12RM × 2-4sets)
Complex pair 3	Weight-bearing Lunge (60%1RM × 8–12RM × 2sets)	Weight-bearing Lunge (65–70%1RM × 8–12RM × 3sets)	Weight-bearing Lunge (80%1RM × 6–12RM/leg×4-8sets)
Complex pair 4	Weight-bearing Heel-lifting (60%1RM × 8–12RM × 2sets)	Weight-bearing Heel-lifting (65–70%1RM × 8–12RM × 3sets)	Weight-bearing Heel-lifting (80%1RM × 6–12RM × 2-4sets)
Complex pair 5	Leg Press (60%1RM × 8–12RM × 2sets)	Leg Press (65–70%1RM × 8–12RM × 3sets)	Leg Press (80%1RM × 6–12RM × 2–4sets)
Rest	Pairs:3 min rest interval Between groups:3–4 min rest	Pairs:3 min rest interval Between groups:3–4 min rest	Pairs:3 min rest interval Between groups:3–4 min rest

**Notes.**

RM: maximum repetitions; % 1RM, percentage of 1RM maximum load intensity.

A concise comparison of the two training programs is provided in Table 4. All sessions were conducted under the direct supervision of two certified strength and conditioning coaches. Attendance was recorded at each session, and participants were required to complete at least 90% of all scheduled sessions to be included in the final analysis. Training loads and repetitions were documented for every session to ensure compliance with the prescribed protocol. Coaches provided real-time feedback on technique and encouraged adherence throughout the intervention.

**Table 4:** Summary of training protocols for CT and RT groups.

**Parameter**	**CT group**	**RT group**
Training frequency	2 sessions/week (Mon & Fri)	2 sessions/week (Mon & Fri)
Duration	60 min/session	60 min/session
Program length	12 weeks	12 weeks
Structure	5 pairs (resistance + plyometric)	Resistance only
Sets per exercise	3	3
Rest between paired exercises	3 min	N/A
Rest between pairs	4 min	N/A
Load adjustment	1RM reassessed every 4 weeks	1RM reassessed every 4 weeks
Exercise selection	Squat + jump squat; deadlift + bounding; lunge + split jump; calf raise + ankle hops; hip thrust + broad jump	Squat, deadlift, lunge, calf raise, hip thrust
Progression	Progressive overload	Progressive overload

### Statistical analysis

The experimental data were processed using JASP (version 0.18.3, JASP Team, The Netherlands). Data are presented as mean ± standard deviation (M ± SD). The normality of the data was assessed using the Shapiro–Wilk test. For normally distributed data, one-way ANOVA was used to examine baseline differences in demographics (age, weight, height, BMI) and outcomes (1RM squat, IMTP, CMJ, SJ, PSAP, and RSI). To evaluate the effects of the intervention on primary outcomes (1RM squat, IMTP, CMJ, SJ, PSAP, and RSI), two-way repeated measures ANOVA was applied. The dependent variables for each model were the primary outcomes, with the independent variables being group (CT and RT), time (pre- and post-intervention), and their interaction. When a significant interaction was detected, Bonferroni *post-hoc* comparisons were performed to identify where the significance occurred. Partial eta squared (*η*^2^) was calculated to determine the effect size for ANOVA interactions, with values of 0.01, 0.06, and 0.14 considered to represent small, medium, and large effects, respectively. Cohen’s d (d) was used to assess the effect size, with classifications as trivial (*d* < 0.2), small (0.2 ≤*d* ≤ 0.6), moderate (0.6 ≤*d* ≤ 1.2), large (1.2 ≤*d* ≤ 2.0), or very large (*d* > 2.0) (Cohen, 2013). The significance level was set at *p* < 0.05.

## Results

All the participants completed this study, and all the data were included in the analysis. All the data were normally distributed (*P* > 0.500). No significant difference in the demographics (*i.e.,* age, body weight, and height), and outcomes measured (*i.e.,* 1RM of squat, IMTP, CMJ, SJ, PSAP, and RSI) were observed between CT and RT group at the baseline(*P* = 0.162).

**Figure 3:**
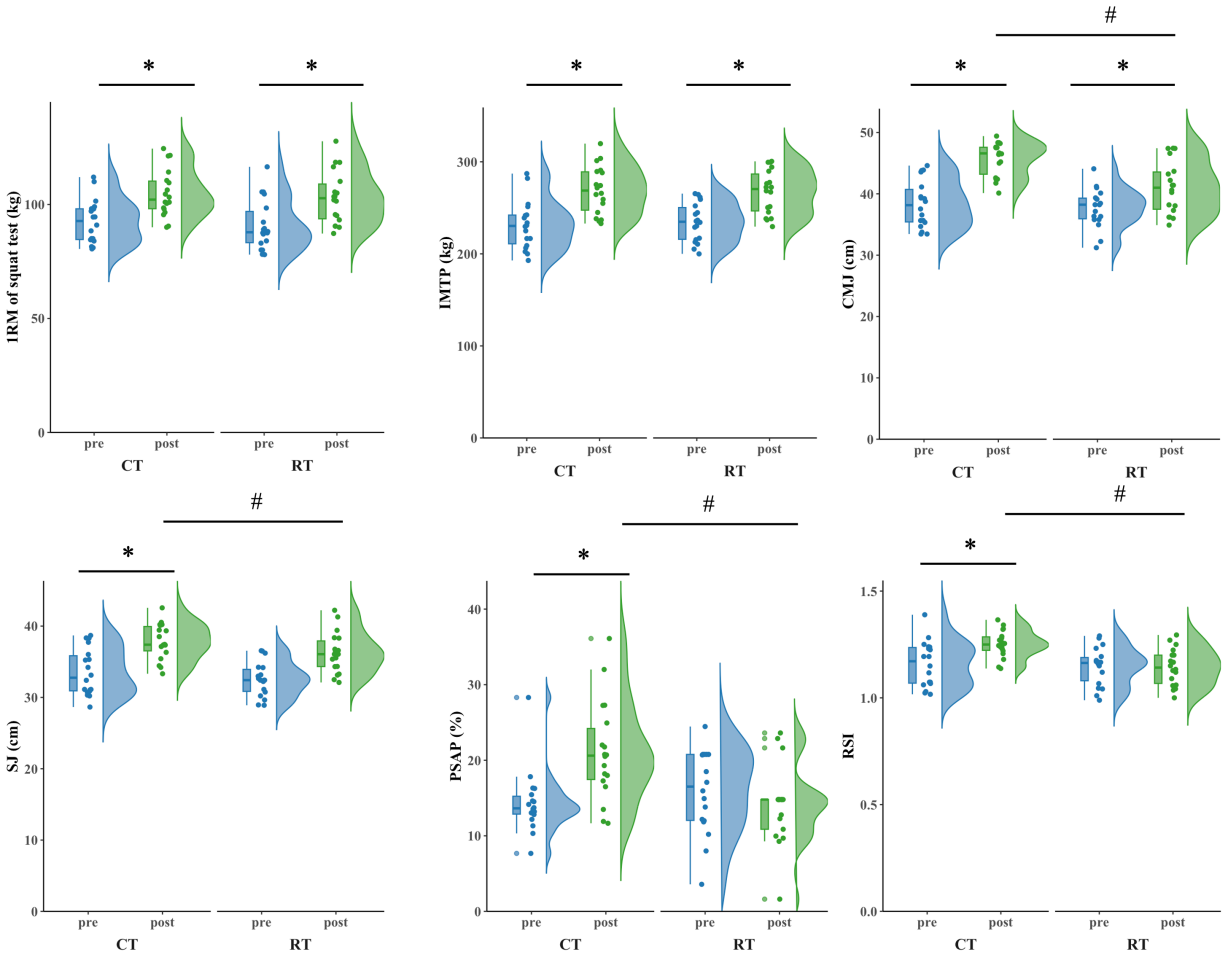
The strength and performance before and after Training. * Statistically significant difference between pre-and pos*t*-test, *p* < 0.05.# Statistically significant difference between group, *p* < 0.05.

The primary two-way repeated-measures ANOVA models showed significant main effects of time (*P* < 0.017), and interactions between group and time on CMJ (*P* < 0.001), PSAP (*P* < 0.001), or RSI (*P* = 0.009). The post-hoc analysis revealed that CMJ (F (1,34) =11.31, *P* < 0.001, partial *η*2 = 0.366, percentages = 19.36%), PSAP (F (1,34) =15.09, *P* < 0.001, partial *η*2 = 0.307, percentages = 31.60%), and RSI (F (1,34) =7.795, *P* = 0.009, partial *η*2 = 0.187, percentages = 7.2%) in CT group were significantly greater after the intervention compared to all the other pre- and post-interventions. Within CT group, CMJ (*P* < 0.001), PSAP (*P* < 0.001), and RSI (*P* = 0.004) were significantly improved after intervention as compared to baseline. Within RT group, CMJ (*P* < 0.001) was significantly improved after intervention as compared to baseline (Table 5 and Fig. 3).

**Table 5:** The assessment results for CT group and RT group before and after 12-week training.

Variable	CT (*N* = 18)			RT (*N* = 18)			*p*-value		
	Pre	Post	Cohen’s d	Pre	Post	Cohen’s d	Time	Group	Time ^*^ Group
1RM of squat (kg)	95.53 ± 9.60	104.84 ± 10.28^*^	1.156	90.81 ± 11.00	103.03 ± 11.59^*^	1.148	0.001^*^	0.615	0.946
IMTP (kg)	231.15 ± 26.33	269.27 ± 25.97^*^	1.563	234.27 ± 21.01	267.09 ± 23.91^*^	1.345	<0.001^*^	0.952	0.315
CMJ (cm)	38.33 ± 3.82	45.75 ± 2.75^*^	2.098	37.71 ± 3.15	41.02 ± 4.26^*^	0.936	<0.001^*^	0.019^*^	<0.001^#^
SJ (cm)	33.52 ± 3.28	37.85 ± 2.50^*^	1.557	32.53 ± 2.39	36.23 ± 2.86^*^	1.332	<0.001^*^	0.107	0.521
PSAP (%)	14.37 ± 4.19	21.01 ± 6.53^*^	1.176	13.17 ± 6.31	15.95 ± 5.53	0.487	0.117	0.037^*^	<0.001^#^
RSI	1.16 ± 0.11	1.25 ± 0.06^*^	1.081	1.15 ± 0.09	1.14 ± 0.09	0.058	0.017^*^	0.015^*^	0.009^#^

**Notes.**

* Significant improvement within group

# Significant improvement between group.

In addition, the exploratory one-way ANOVA models showed that within both CT and RT group, 1RM of squat (*P* < 0.001), IMTP (*P* < 0.001), and SJ (*P* < 0.001) were significantly improved after the intervention as compared to baseline.

## Discussion

To the best of our knowledge, this is the first study to explore the effect of a 12-week complex training program on lower limb strength and power in collegiate dancers. Given that most dancers, including those in our sample, have limited prior exposure to systematic strength training, it was initially unclear whether CT would produce the same benefits observed in other athletic populations. The results of present study indicate that both CT and RT could exhibit significant improvements in strength and power in collegiate dancers. However, CT demonstrated superior efficacy in enhancing measures of lower limb power, including CMJ, PSAP, and RSI, compared to RT. These findings suggest that CT may serve as an effective training method for boosting strength and power in collegiate dancers, potentially enhancing their dance performance’s ability is foundational for movement like leaps and turns that require height, control, and sustained power.

Enhanced CMJ ability allows dancers to achieve more dynamic and visually impactful performances, making it a valuable measure of lower-limb power that directly translates to aesthetic and technical performance in dance. However, it should be noted that this implication is inferred from improvements in physical performance measures, as no direct assessments of dance-specific technical skills or artistic quality were conducted in this study. Therefore, these potential links between CMJ gains and actual dance performance should be interpreted with caution. Our results showed that following both CT and RT program, there was a significant improvement in CMJ. However, the improvement in CT was significantly greater than RT. The results agree with previous research in some other sport athlete (Hammami et al., 2019; Thapa et al., 2021; Kobal et al., 2017). Hammami et al. (2019) demonstrated that CT could increase the jump and sprint performance in junior female handball players. Kobal et al. (2017) also showed the combination of plyometric and resistance training could significantly enhance the maximum dynamic strength and jump performance. This enhanced effect can be partially attributed to the unique combination of strength and plyometric exercises in CT, which optimizes neural activation through mechanisms like post-activation potentiation (PAP) (Drouzas et al., 2020). Studies such as those by Drouzas et al. (2020) show that the inclusion of plyometric exercises in CT enhances neuromuscular coordination and muscle activation, creating an optimal environment for explosive power development. These neural adaptations likely play a significant role in the superior gains in CMJ observed with CT, as improved neuromuscular function enhances the efficiency and power of explosive movements like jumping (Hammami et al., 2019).

The improvement in PSAP further supports the gains in CMJ, as it reflects enhanced efficiency in the SSC (Kosaka et al., 2023). The significant increase in PSAP, especially in the CT group, indicates that dancers trained under CT have become more adept at utilizing the SSC. This enhanced SSC efficiency means that dancers can generate more force from the same muscle activation, directly translating to greater jump height in CMJ. Study by Suchomel, Sole & Stone (2016) support this by demonstrating that plyometric exercises improve SSC responsiveness, amplifying the benefits seen in CMJ through more efficient energy use. Thus, while strength gains from both CT and RT contribute to CMJ improvements, CT’s superior effect on PSAP likely amplifies the observed benefits in CMJ, making it a more effective training method for maximizing jump performance through both muscular and elastic components.

RSI, which measures the ability to quickly generate force after ground contact (Southey et al., 2024), complements the CMJ by indicating an improved ability to handle repeated, high-impact movements with minimal recovery time. This capability is crucial for sequences that involve successive jumps or quick transitions, where maintaining momentum and minimizing delay are essential (Pedley et al., 2022). In conjunction with CMJ, RSI improvements reflect not only a dancer’s ability to jump higher but also their ability to recover and quickly transition to subsequent movements. This combination is particularly valuable in dance, where smooth and rapid transitions are fundamental to fluid choreography. The superior outcomes observed with CT in CMJ, PSAP, and RSI are likely attributable to its inherent combination of heavy resistance exercises and plyometric drills, which capitalizes on PAP. Additionally, the plyometric component improves SSC efficiency by increasing tendon stiffness, enhancing elastic energy storage, and refining neuromuscular coordination (Hirayama et al., 2017). Together, these adaptations increase the rate of force development and enable more effective use of stored elastic energy, leading to greater jump height and faster ground contact times. Such mechanisms are particularly beneficial in dance, where repeated explosive movements, rapid transitions, and controlled landings are essential for performance quality.

The lack of significant differences between CT and RT in improving IMTP, 1RM and SJ highlights that both methods are equally effective in enhancing maximal strength. SJ, IMTP and 1RM primarily assess concentric strength without involvement of the SSC (Nishioka & Okada, 2022). These findings are consistent with previous research conducted by Talpey, Young & Saunders (2016), who have shown that CT were comparable to RT in terms of improving maximal strength. This equivalence may be explained by the fact that both programs incorporated similar resistance training components and total training volume for strength development, which are primary drivers of maximal force gains, and thus may have produced comparable adaptations in maximal strength despite the additional plyometric component in CT. These results indicate that while CT incorporates a plyometric component to improve explosive power *via* SSC, it does not provide a significant advantage over RT in terms of pure strength gains.

## Conclusion

CT significantly improved both strength and explosive power in collegiate dancers, with superior effects on jump-related outcomes compared with RT. This approach offers a time-efficient and practical method to enhance dancers’ physical performance.

## Limitations

This study has several limitations. First, due to the limited methods available for assessing dance-specific abilities, this research did not directly measure improvements in dance-specific performance, so translation to artistic performance remains speculative. Future studies could benefit from developing or incorporating assessments that more accurately reflect the practical impact of training on dance skills. Second, participants in this study were male, whereas female dancers represent a larger proportion of the dance population. Further research is needed to verify the effectiveness of CT in female dancers to ensure the generalizability of these findings across genders. Moreover, while a correlation analysis between changes in strength/power measures and potential performance indicators could have provided additional insight, this was not performed due to the relatively small sample size and the primary aim of the study being to compare group-level training effects rather than individual-level associations. Future studies with larger cohorts should include such analyses to explore the relationships between neuromuscular adaptations and specific performance outcomes in dancers. Additionally, while our findings suggest that the integration of resistance and plyometric exercises in CT contributes to enhanced neural activation, it is possible that similar neuromuscular adaptations could be elicited through plyometric training alone. As this study did not include a plyometric-only group, future research should compare CT directly with isolated plyometric training to determine whether the observed neural benefits are unique to CT or can be achieved through plyometrics alone.

## Practical Implications

Based on the findings of this study, dance educators and coaches can consider incorporating complex training into regular training programs as a supplement to traditional dance practice. CT can be scheduled twice per week, combining heavy resistance exercises (*e.g.*, squats, lunges) with plyometric drills (*e.g.*, jump squats, box jumps) in the same session. This combined format makes CT time-efficient and practical, especially for collegiate dancers who face tight schedules due to academic and rehearsal demands. This approach can improve lower-limb power and strength, helping dancers achieve higher jumps, faster movements, and better overall performance.

## Supplemental Information

10.7717/peerj.20486/supp-1 Supplemental Information 1 Raw data

10.7717/peerj.20486/supp-2 Supplemental Information 2 Warm-up, Cool-down, and Familiarization Procedures

10.7717/peerj.20486/supp-3 Supplemental Information 3 Translations for the Chinese text in the raw data

## Additional Information and Declarations

### Data Availability

The following information was supplied regarding data availability: The raw data is available in the Supplemental Files.
