# Supplementary material for: Effects of 12 weeks of complex training on lower limbs strength and power in collegiate dancers
Source: PeerJ. 2026 May 21;14:e20486. doi: 10.7717/peerj.20486 (PMC13198847; doi:10.7717/peerj.20486)
Supplement: Supplemental Information 2 [file peerj-14-20486-s002.docx]

**Table S1. Warm-up, Cool-down, and Familiarization Procedures**

| Procedure | Description |
| --- | --- |
| Warm-up | Each training session began with a standardized 10-min dynamic warm-up consisting of: • 5 min of low-intensity running (jogging, high-knee skipping, lateral shuffles, arm circles) • 5 min of dynamic stretching targeting the lower limbs (e.g., walking lunges, leg swings, hip openers) |
| Cool-down | After each session, participants performed: • 5 min of low-intensity jogging or walking • 5–10 min of static stretching focusing on the quadriceps, hamstrings, calves, glutes, and hip flexors |
| Familiarization | Prior to the experimental training program, participants completed two familiarization sessions to ensure proper technique and reduce learning effects during testing procedures. |
