# Supplementary material for: Effects of 12 weeks of complex training on lower limbs strength and power in collegiate dancers
Source: PeerJ. 2026 May 21;14:e20486. doi: 10.7717/peerj.20486 (PMC13198847; doi:10.7717/peerj.20486)
Supplement: Supplemental Information 3 [file peerj-14-20486-s003.docx]

CT 组：CT Group
RT组：RT Group
